# Mesenchymal‐epithelial transition factor exon 14 skipping mutation‐positive granulocyte colony‐stimulating factor‐producing lung adenocarcinoma mimicking lung abscess: A case report

**DOI:** 10.1002/rcr2.1419

**Published:** 2024-06-25

**Authors:** Yuka Izumiya, Hidesato Odaka, Toru Kikuchi, Yuri Takita, Takuo Tokairin

**Affiliations:** ^1^ Department of Respiratory Medicine Japanese Red Cross Akita Hospital Akita Japan; ^2^ Post Graduate Clinical Education Center Japanese Red Cross Akita Hospital Akita Japan; ^3^ Department of Pathology Japanese Red Cross Akita Hospital Akita Japan

**Keywords:** G‐CSF‐producing tumour, lung abscess, lung adenocarcinoma, MET exon 14, sterile lung abscess, tepotinib

## Abstract

Granulocyte colony‐stimulating factor (G‐CSF)‐producing lung tumours are rare, with their imaging features and effective treatments remaining elusive. Similarly, mesenchymal‐epithelial transition (MET) exon 14 skipping mutations are also uncommon. Herein, we report a case of G‐CSF‐producing lung adenocarcinoma positive for a MET exon 14 skipping mutation, mimicking lung abscess. A 61‐year‐old man presented with cough and high fever. Contrast‐enhanced chest computed tomography revealed a mass with a cavity and internal fluid accumulation. The patient initially underwent diagnostic treatment for a lung abscess but was ultimately diagnosed with lung adenocarcinoma positive for a MET exon 14 skipping mutation. Following tepotinib therapy, the primary lesion shrank, and serum G‐CSF levels decreased, leading to a diagnosis of G‐CSF‐producing lung cancer. G‐CSF‐producing lung tumours can present imaging findings that mimic lung abscesses. Tepotinib therapy may be effective for patients with MET exon 14 skipping mutation, including those with G‐CSF‐producing lung cancer.

## Graphical abstract

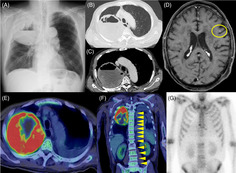

## INTRODUCTION

Granulocyte colony‐stimulating factor (G‐CSF) is a cytokine that stimulates the bone marrow to produce granulocytes and stem cells that are released into the bloodstream. G‐CSF‐producing lung tumours are rare and generally exhibit a progressive clinical course with a poor prognosis. Currently, the imaging features of these tumours are not well understood, and effective treatments have yet to be identified.

The mesenchymal‐epithelial transition factor (MET) is an oncogene that encodes a tyrosine kinase receptor. MET exon 14 skipping mutations have been reported to activate tumorigenesis and are found in 2–4% of non‐small cell lung cancer (NSCLC) cases.^1^ Although rare, these mutations have shown responsiveness to MET inhibitors, which are effective in patients with NSCLC who exhibit high levels of MET amplification.

Herein, we report a case of G‐CSF‐producing lung adenocarcinoma positive for MET exon 14 skipping mutation mimicking lung abscess.

## CASE REPORT

**FIGURE 1:**
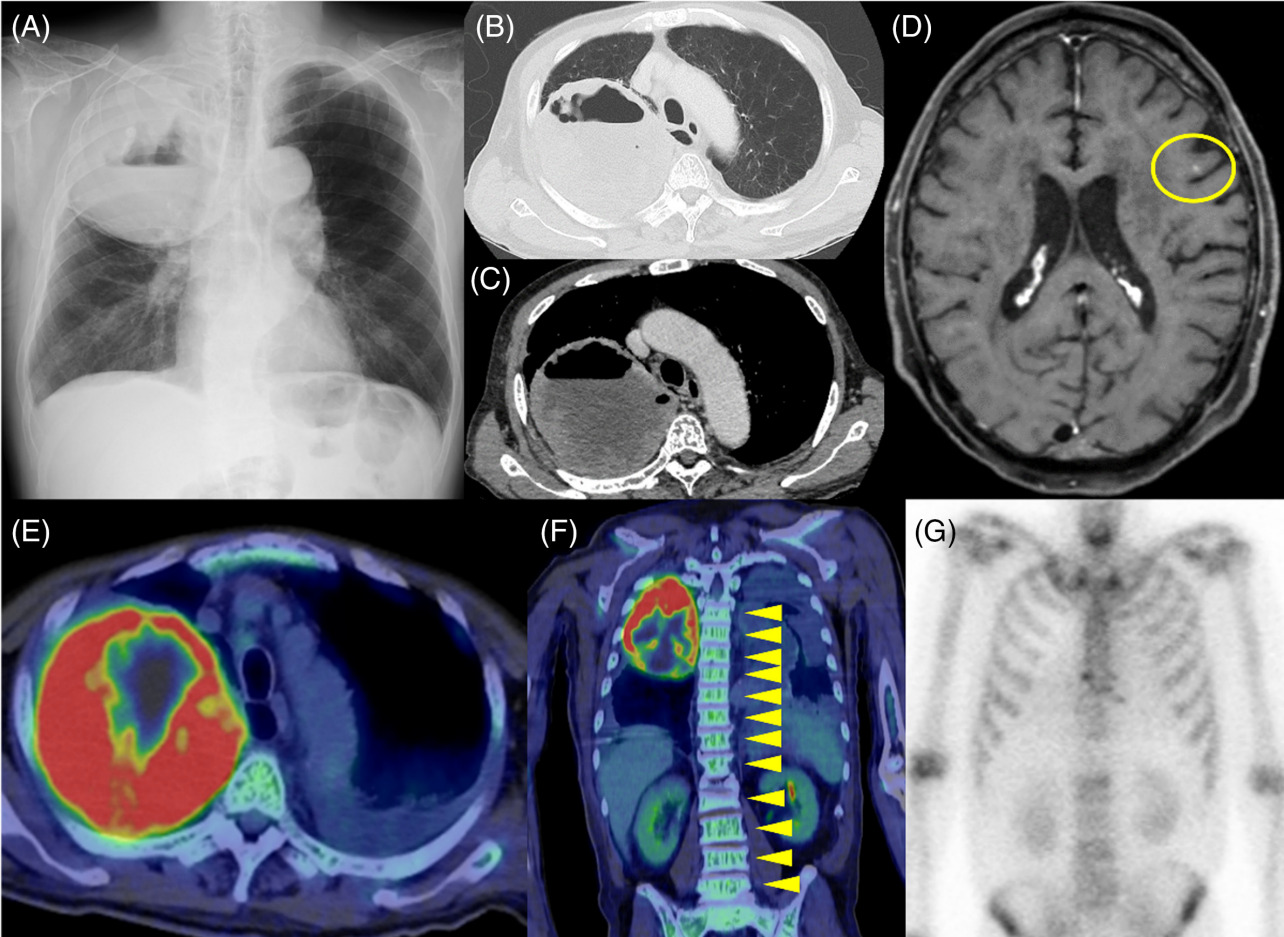
Image inspection on admission. (A) Chest radiograph revealed an 11 cm tumour shadow in the upper right lung field. It was formed with a cavity. (B) and (C) Chest contrast‐enhanced computed tomography revealed a mass in the right upper lobe. It was formed with a cavity and internal liquid storage. (D) Magnetic resonance imaging T2‐weighted images showed a tumour in the left frontal lobe (yellow circle). (E) 18F‐fluorodeoxyglucose positron emission tomography/computed tomography (18F‐FDG‐PET/CT) revealed elevated uptake in the mass in the right upper lobe, with a maximum standardized uptake value max of 18.0. (F) 18F‐FDG‐PET/CT revealed diffuse uptake in the bone marrow (yellow arrowheads). (G) Bone scintigraphy revealed no significant uptake in the spine.

A 61‐year‐old man presented with cough and high fever (body temperature: 38°C) persisting for approximately 1 month. Blood tests revealed an elevated white blood cell count of 37,400/μL, with a neutrophil count of 35,500/μL, and a high C‐reactive protein level of 11.4 mg/dL. Contrast‐enhanced chest computed tomography (CT) revealed an 11 cm mass in the right upper lobe of the lungs, characterized by a cavity and internal fluid accumulation (Figure 1A–C).

The patient was tentatively diagnosed with a lung abscess based on imaging and inflammatory findings, and symptoms. He was treated with sulbactam/ampicillin (12 g/day) for 5 days, followed by meropenem (3 g/day) for 10 days. However, there was no improvement, leading to the suspicion of a lung tumour.

**FIGURE 2:**
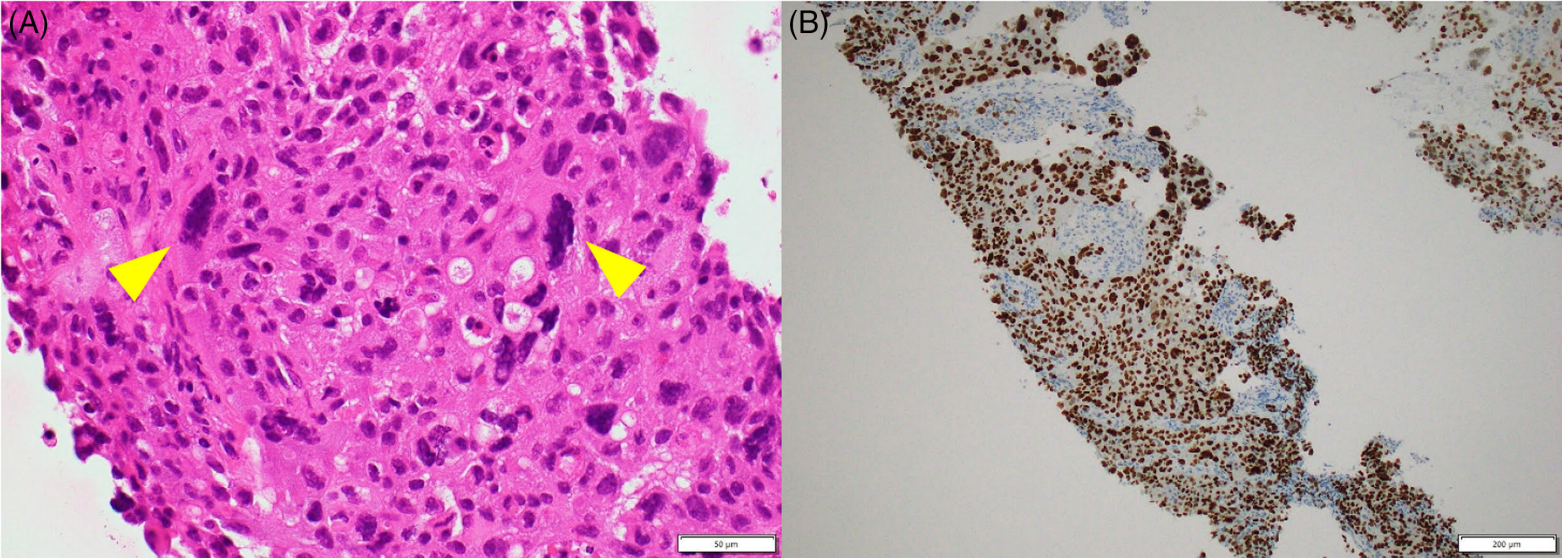
Histopathological findings of the biopsy specimen from the lung tumour. (A) Haematoxylin and eosin staining revealed atypical epithelial cells with giant cells arranged in a sheet (yellow arrowheads) (×400). (B) Immunohistochemistry showed that the tumour cells were positive for thyroid transcription factor‐1 (×100).

A CT‐guided lung biopsy was performed on the tumour in the right lung. The tissue revealed atypical epithelial cells with giant cells and poorly differentiated NSCLC cells. Immunohistochemistry indicated that the tumour cells were positive for thyroid transcription factor‐1, leading to a diagnosis of adenocarcinoma (Figure 2A,B). Magnetic resonance imaging T2‐weighted images revealed a 3 mm tumour in the left frontal lobe, suggestive of brain metastasis (Figure 1D). Additionally, 18F‐fluorodeoxyglucose positron emission tomography/CT (FDG‐PET/CT) revealed elevated uptake in the mass in the right upper lobe, with a maximum standardized uptake value max of 18.0 (Figure 1E). Diffuse uptake in the bone marrow was also identified (Figure 1F). Bone scintigraphy revealed no significant uptake in the spine (Figure 1G). The disease was staged as cT4N0M1c, stage IVB. No bacteria were detected in sputum, blood, or biopsy tissue cultures.

Since the tumour tissue was positive for MET exon 14 skipping mutation, we commenced treatment with tepotinib at 500 mg/day. A decrease in tumour size was observed following the initiation of therapy, and the patient remained progression‐free for 6 months. Although the patient's serum G‐CSF levels were initially elevated at approximately 165.0 pg/mL (normal range: <39.0 pg/mL), it decreased to 28.4 pg/mL 35 days after starting treatment. Based on these findings, the patient was diagnosed with G‐CSF‐producing lung cancer.^2^

## DISCUSSION

Two significant clinical observations were made in this case report. First, G‐CSF‐producing lung tumours can present as sterile lung abscesses. In the present case, chest CT revealed image findings similar to those of a lung abscess. A review of literature identified 24 published articles on G‐CSF‐producing lung tumours. In 23 of these cases, the primary lesion on the chest CT exhibited a low‐attenuation area.^3^ Furthermore, the image findings of the cases resembled those of lung abscesses. Although the exact mechanism remains unknown, it is speculated that hypersecretion of G‐CSF from the aggressive tumour results in neutrophil infiltration, forming abscesses.^4^ While lung abscesses are typically infectious, the 23 cases mentioned above, as well as the present case, had sterile lung abscesses. Additionally, a case of G‐CSF‐producing gastric cancer reported multiple sterile brain and lung abscesses upon autopsy, without bacteriological signs.^4^ This suggests that G‐CSF‐producing lung cancers can lead to sterile lung abscesses. However, further accumulation of cases is necessary to confirm this observation. If it is clarified that G‐CSF‐producing lung tumours can present imaging findings similar to lung abscesses, earlier diagnosis of G‐CSF‐producing lung cancer may be possible, potentially avoiding unnecessary antibiotic use. Feki et al. suggests reconsidering the diagnosis of lung abscess when there is no improvement after 10 days of antibiotic treatment.^5^

In the present case, diffuse uptake in the bone marrow was identified, despite bone scintigraphy revealing no significant uptake in the spine. Miyako et al. reported that diffuse uptake in the bone marrow is a characteristic finding of G‐CSF‐producing tumours in FDG‐PET/CT.^6^ This finding may also be useful for the earlier diagnosis of G‐CSF‐producing lung cancer.

The second clinical observation was that tepotinib therapy can be effective in patients with MET exon 14 skipping mutations, including those with G‐CSF‐producing lung cancer. Clinical trials of tepotinib have demonstrated the efficacy of this agent in patients with advanced NSCLC with a confirmed MET exon 14 skipping mutation.^7^ However, its effect on G‐CSF‐producing lung cancer has not been previously reported. In our case, tepotinib showed sufficient efficacy.

In conclusion, G‐CSF‐producing lung tumours can manifest as sterile lung abscesses, and tepotinib therapy may effectively target these tumours in patients with MET exon 14 skipping mutations, even in cases of G‐CSF‐producing lung cancer.

Further studies are warranted to determine whether lung cancers presenting with imaging findings similar to those of lung abscesses are more prevalent. Additionally, research is needed to elucidate the mechanism underlying the formation of lung abscesses in these cases.

## AUTHOR CONTRIBUTIONS

*Conceptualization*: Yuka Izumiya, Hidesato Odaka; data curation, Yuka Izumiya, Toru Kikuchi. *Investigation*: Yuka Izumiya, Toru Kikuchi, Yuri Takita. *Supervision*: Hidesato Odaka; *Visualization*: Yuka Izumiya, Toru Kikuchi, Yuri Takita, Takuo Tokairin. *Writing – original draft*: Yuka Izumiya, Hidesato Odaka. *Writing – review and editing*: Hidesato Odaka, Takuo Tokairin.

## ETHICS STATEMENT

The authors declare that appropriate written informed consent was obtained for the publication of this manuscript and accompanying images.

## Data Availability

The data that support the findings of this study are available on request from the corresponding author. The data are not publicly available due to privacy or ethical restrictions.
